# Novel Therapeutic GPCRs for Psychiatric Disorders

**DOI:** 10.3390/ijms160614109

**Published:** 2015-06-19

**Authors:** Hidetoshi Komatsu

**Affiliations:** Novartis Pharma, Medical Division, CNS Medical Franchise Department, Tokyo 105-6333, Japan; E-Mail: hidetkomatsu@fuji.waseda.jp; Tel.: +81-3-6899-7155; Fax: +81-3-6257-3620

**Keywords:** psychiatric disorders, striatum, schizophrenia, GPCR, GPR52, dopamine receptors, GPR88, GPR6

## Abstract

G protein-coupled receptors (GPCRs) are the most common targets of the neuropharmacological drugs in the central nervous system (CNS). GPCRs are activated by manifold neurotransmitters, and their activation in turn evokes slow synaptic transmission. They are deeply involved in multiple neurological and psychiatric disorders such as Parkinson’s disease and schizophrenia. In the brain, the striatum is strongly innervated by the ventral tegmental area (VTA) and plays a central role in manifestation of psychiatric disorders. Recently, anatomical and comprehensive transcriptome analysis of the non-odorant GPCR superfamily revealed that the orphan GPCRs GPR88, GPR6, and GPR52, as well as dopamine D1 and D2 receptors and the adenosine A2a receptor, are the most highly enriched in the rodent striatum. Genetically engineered animal models and molecular biological studies have suggested that these striatally enriched GPCRs have a potential to be therapeutic psychiatric receptors. This review summarizes the current understanding of the therapeutic GPCR candidates for psychiatric disorders.

## 1. Introduction

Approximately 60% of all drugs target membrane proteins. Among them, G protein-coupled receptors (GPCRs), also known as seven-transmembrane domain proteins, are one of the most important classes of therapeutic targets and account for around 30% of all current drug targets. GPCRs constitute the largest receptor superfamily and control myriad physiological and disease-signaling processes. GPCRs can be divided to two groups: odorant/sensory and non-odorant. Odorant/sensory receptors are confined to the sensory cells, including olfactory neurons, taste cells, and photoreceptor cells that detect external stimuli such as light, odors, tastes, and pheromones. Non-odorant receptors are expressed throughout the whole body and detect a variety of ligands. They govern numerous physiological responses including hemostasis, immune function, reproduction, cardiac function, metabolism, and neurotransmission [1]. Therefore, non-odorant GPCRs can be considered the most promising drug targets.

Neurotransmitters exert their functions through two classes of receptors that possess distinctive modalities of synaptic transmission. Ionotropic receptors comprise the ligand-gated ion channels that elicit fast synaptic transmission. In contrast, metabotropic receptors consist of GPCRs that bind to neurotransmitters and cause slow synaptic transmission through intracellular signaling pathways as well as induction of gene expression necessary for exerting antipsychotic actions [2,3,4]. Notably, most neuropharmacological drugs are known to regulate GPCR activity in the central nervous system (CNS) [5].

Dopamine receptors represent archetypal examples of GPCRs mediating neurotransmission. Dopamine is a monoamine neurotransmitter responsible for reward, locomotion, and affection. Defects in dopaminergic neurotransmission lead to multiple neurological and psychiatric disorders such as Huntington’s disease, attention deficit hyperactivity disorder (ADHD), mood disorders, Parkinson’s disease, and schizophrenia. It has been demonstrated that GPCRs are deeply involved in mitigating symptoms of schizophrenia, since typical and atypical antipsychotics such as haloperidol and olanzapine have antagonistic activities for the dopamine D2 receptor as well as other multiple GPCRs.

In general, GPCR transcripts are expressed at low levels and often fail to be measured precisely by DNA microarray analysis. Indeed, GPCR transcripts account for only 0.001%–0.01% of the expressed sequence tags (ESTs), although non-odorant GPCRs constitute about 1% of genes in the whole genome [6]. In recent years, Regard *et al.* and Komatsu *et al.* revealed comprehensive transcript profiling of non-odorant GPCR family throughout C57BL/6 mouse tissues via quantitative real-time PCR (qPCR) [7,8]. Furthermore, Komatsu *et al.* identified novel neurotherapeutic GPCR candidates exclusively expressed in the brain, especially in the striatum, which is closely associated with psychiatric disorders such as schizophrenia [8]. They found that GPR6, GPR52, and GPR88, known as orphan GPCRs, co-localize either with the dopamine D2 receptor alone or with both the dopamine D1 and D2 receptors in neurons of the basal ganglia, and propose that among these orphan receptors, GPR52 has the highest potential of being a therapeutic psychiatric receptor. This review summarizes the current understanding of the potential therapeutic GPCRs for psychiatric disorders as well as their anatomical expression profiles.

## 2. GPCRs in CNS

Diverse members of non-odorant GPCR superfamily have been reported to consist of 367 receptors in humans and 392 in mice where 343 are common to the two species, based on extensive analysis of public human and mouse genome sequence databases [9]. Non-odorant GPCRs, approximately one fourth of which are orphan receptors, are abundantly expressed in the CNS, especially in the brain [8,9]. Regard *et al.* proposed that more than 80% of the 353 non-odorant GPCRs are expressed in mouse CNS. Komatsu *et al.* found that there are 6 clusters of GPCRs that exhibit rich and relatively specific mRNA expression in the CNS, which account for approximately 40% of the 322 non-odorant GPCRs in mice. For example, dopamine, serotonin, glutamate, and acetylcholine receptors, all of which are well-known neuropharmacological targets, are included in these 6 clusters and highly expressed in the brain. These findings suggest that the brain-specific non-odorant GPCRs have great potential as therapeutic targets for CNS drugs.

## 3. Medium-Sized Spiny Neurons (MSNs) in the Striatum Control Psychiatric Symptoms

The striatum is critically involved in a variety of neuro-psychiatric diseases such as addiction, Tourette’s syndrome, Parkinson’s and Huntington’s diseases, attention deficit hyperactivity disorder (ADHD), and schizophrenia, and is the major input structure of the basal ganglia [10]. Dopamine modulates how this input is processed in the striatum where dopamine D1 and D2 receptors are most abundant [11]. The striatum is strongly innervated by the ventral tegmental area (VTA) and the substantia nigra pars compacta (SNc). The VTA is the origin of the dopaminergic mesolimbic pathway, which is known to be overactive in schizophrenia [10,12].

The rodent striatum is comprised of about 95% GABAergic medium-sized spiny neurons (MSNs) and 5% of interneurons including large aspiny cholinergic neurons [13,14,15]. MSNs constitute the striatonigral (direct) and striatopallidal (indirect) pathways and can be divided into two types of neuronal populations based on their projections and the receptors and neuropeptides they express [16]. These two MSN populations are morphologically very similar and distributed across the striatum. In the direct pathway, the striatonigral neurons project onto the substantia nigra pars reticulata (SNr) and the medial globus pallidus (MGP). The striatonigral neurons specifically express neuropeptides substance P and dopamine D1 receptors [17,18]. In the indirect pathway, the striatopallidal neurons project onto the lateral globus pallidus (LGP). This pathway reaches the SNr/MGP via the subthalamic nucleus (STN). The striatopallidal neurons specifically express neuropeptide enkephalin, dopamine D2 receptors, and adenosine A2A receptors [17,18,19,20].

According to a proposed model of the basal ganglia, the striatonigral (direct) and striatopallidal (indirect) pathways have opposite but balancing roles in regulating the motor behavior [21]. In this model, the direct pathway facilitates locomotion whereas the indirect pathway abrogates movement [22]. MSNs are also deeply involved in reward, motivation, and addiction, as well as manifestation of Parkinson’s disease and schizophrenia [10,11], but their differential functions still remain elusive.

## 4. Antipsychotics Exert Therapeutic Action through Dopamine D1 and D2 Receptors

Dopamine signaling plays a key role in schizophrenia, since all commonly prescribed antipsychotics have an antagonistic activity against the Gi/o-coupled dopamine D2 receptor, which is most enriched in the striatum, where a Gs-coupled dopamine D1 receptor is also highly expressed [11]. Schizophrenia is a severe mental disorder of unknown etiology with complex inheritance patterns, affecting nearly 1% of the population. Its symptoms include positive symptoms (e.g., delusion, hallucination and thought disorder), negative symptoms (e.g., apathy, poor social functioning and emotional blunting), cognitive deficits and other psychopathological symptoms (e.g., psychomotor retardation, lack of insight, poor attention and impulse control) [23]. These schizophrenic symptoms are likely linked not only to hyperactive dopaminergic transmissions in the mesolimbic pathway (from the VTA to the limbic system’s nucleus accumbens, a collection of neurons in the striatum) but also to the decreased dopamine release in the prefrontal cortex. Blocking dopamine D2 receptors with any antipsychotic drug in the mesolimbic system is thought to alleviate positive symptoms but not sufficiently address negative symptoms and cognitive deficits.

Existing antipsychotics can be generally divided into two categories, typical (first-generation) antipsychotics and atypical (second-generation) antipsychotics. Typical antipsychotics such as haloperidol mainly possess an antagonistic activity for dopamine D2 receptor(s), whereas atypical antipsychotics such as olanzapine and clozapine show antagonistic activities for serotonin 2A and dopamine D2 receptors and multiple other GPCRs [24]. Typical antipsychotics are more likely to induce some unwanted side effects such as extrapyramidal side effects (EPS) and hyperprolactinaemia than atypical antipsychotics. Aripiprazole, classified as an atypical antipsychotic, is a partial dopamine D2 receptor agonist whilst it acts as an antagonist for the D2 receptor in the state of excessive dopaminergic neurotransmission [25,26]. The atypical antipsychotic clozapine has a unique property distinguished from other antipsychotic drugs by virtue of its higher affinities for dopamine D1 and 5-HT 2A receptors and its lower affinity for D2 receptors [24,27]. In schizophrenia, D2 receptor signaling is considered to be overactivated [28], whereas the D1 receptor is reduced in the prefrontal cortex in correlation with the severity of the negative symptoms and cognitive deficits [29].

## 5. Striatal-Enriched GPCRs Are Potential Drug Targets for Psychiatric Disorders

**Figure 1:**
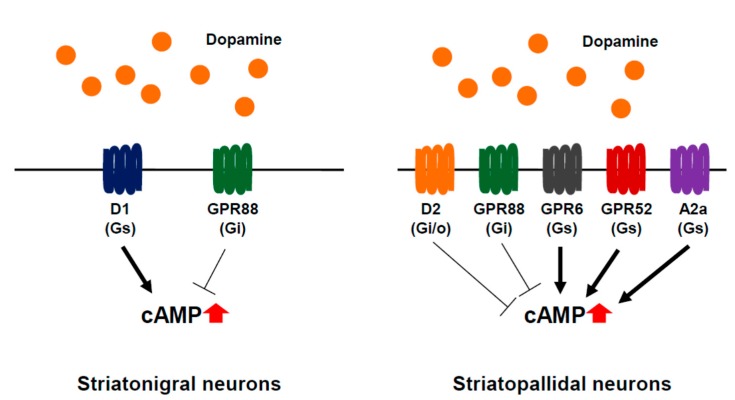
Striatal-enriched GPCRs in medium-sized spiny neurons (MSNs) in striatum. MSNs can be divided into two types of neurons: striatonigral (**left**) and striatopallidal MSNs (**right**). Gs-coupled receptors including D1, GPR52, GPR6, and A2a receptors raise intracellular cAMP concentration which can be reduced by Gi/o-coupled receptors including D2 and GPR88.

As described above, the striatum is an ideal area of investigation to study potential therapeutic targets of psychiatric disorders. Recently, comprehensive transcriptional analysis of GPCRs has revealed that GPR88, GPR6, and GPR52, as well as dopamine D1 and D2 receptors and adenosine A2a receptor, are the most highly enriched in mouse striatum [8]. GPR88, GPR6, and GPR52 are known as orphan GPCRs and show almost identical expression patterns to those of D1 and D2 receptors in the brain at a transcriptional level. Intriguingly, *in situ* hybridization (ISH) analysis of the basal ganglia indicates that GPR88 is expressed in both striatonigral and striatopallidal neurons expressing D1 and D2 receptors, respectively, whereas GPR52 and GPR6 are expressed in the striatopallidal neurons but not in the striatonigral neurons (Figure 1). In the following section, these three orphan GPCRs as well as A2a receptor are briefly explained.

### 5.1. Adenosine A2a Receptor

Adenosine acts mainly through adenosine A1 and A2a receptors and controls a wide range of brain functions. The A2a receptor is known to affect a wide range of neuropsychiatric functions primarily via dopaminergic and glutamatergic neurotransmission that could be relevant to the potential therapeutic interest of psychiatric disorders, particularly in psychostimulation, drug addiction, anxiety, depression, and psychiatric disorder [30]. The Gs-coupled A2a receptor is predominantly expressed in the striatopallidal MSNs (Figure 1), and plays a pivotal role in the control of motor function since its ligand induces most significant motor effects [20,31]. Dopaminergic neurotransmission modulated by A2a receptor is psychopharmacologically relevant, whereas the control of GABAergic transmission from the striatopallidal MSNs by the A2a receptor antagonists leads to Parkinson’s disease (PD) therapy [32,33]. The mechanism by which A2a antagonists improve motor activity is mediated through modulation of GABA release.

Antagonistic interaction of A2a and D2 receptors has been proposed as the basis for potential therapy of psychiatric illness. A2a agonists inhibit the motor, discriminative, and rewarding effects of psychostimulants, showing an atypical antipsychotic profile in animal models [34,35]. Pharmacological and genetic studies suggest that the A2a receptor activity affects schizophrenia-like behaviors in patients. Non-selective A1 and A2a receptor antagonist caffeine exacerbates positive symptoms of schizophrenia [36,37]. A single-nucleotide polymorphism (SNP) of the A2a receptor is a candidate for a schizophrenia susceptibility gene on chromosome 22q12–13 [38,39].

A number of molecular studies suggest that the A2a receptor functions as a fine-tuner in balancing a glutamatergic-dopaminergic network [40]. In dopaminergic function, the antagonistic interaction of A2a and D2 receptors in striatum suggests antipsychotic behaviors in schizophrenia via an A2a receptor agonist functioning as a dopamine receptor antagonist. A2a receptor activation can diminish D2 receptor affinity and activity, which may underlie the antipsychotic-like profile [41]. In glutamatergic function, A1 and A2a receptor agonists have both been found to prevent electroencephalogram (EEG) and behavioral effects evoked by NMDA receptor antagonists [42,43]. In an NMDA receptor hypofunction model of schizophrenia, the NMDAR function can be regulated by both A1 and A2A receptor activities [44,45]. Importantly, the psychostimulant effects by NMDA receptor antagonists are largely abolished by genetic inactivation or pharmacological blockade of A2a receptors, suggesting that modulation of A2a receptor may restore the hypofunction of NMDA receptors in animal models of schizophrenia [34,46].

### 5.2. GPR88

GPR88, an orphan GPCR, is highly and almost exclusively expressed in both the striatonigral and striatopallidal MSNs of the basal ganglia (Figure 1) [8,47]. GPR88 knockout mice show abnormal behaviors associated with schizophrenia, such as disrupted sensorimotor gating, accentuated behavioral response to apomorphine and amphetamine [48], and impaired learning [47]. The RNA interference-mediated knockdown of GPR88 in rats attenuates the amphetamine-induced hyperlocomotion and reduces the impairment of social novelty discrimination elicited by neonatal exposure to PCP [49]. Electrophysiological investigation of the MSNs in the knockout mice has shown that tonic GABAergic inhibition and responses to synaptically released GABA is attenuated whereas glutamatergic excitatory synaptic transmission is augmented [47]. The phosphorylation of the AMPA-type glutamate receptor GluR1 is increased in the knockout mice, suggesting that GPR88 play a critical role in postsynaptic signaling for the efficacy of glutamatergic transmission [47].

Previous studies have identified GPR88 as a susceptibility gene for both bipolar disorder and schizophrenia by a genetic association analysis [50], and antidepressant treatment increases GPR88 expression [51]. Taken together, these findings suggest that GPR88 may be implicated in the manifestation of psychiatric disorders and has been considered a potential therapeutic target for these diseases.

The signal transduction pathway and receptor functions of GPR88 still remain largely unclear due to the lack of endogenous ligands. Another possibility is that GRP88 may dimerize with non-orphan GPCRs [52]. Recently, the synthesis of a small molecular agonist for GPR88, 2-PCCA, has been reported [53]. 2-PCCA inhibits intracellular cAMP accumulation and fails to elicit calcium influx in cells expressing GPR88, suggesting that GPR88 is coupled to Gi. This discovery of the agonistic compounds will facilitate the understanding of physiological functions of GPR88.

### 5.3. GPR6

GPR6 is predominantly expressed in the striatopallidal neurons in the basal ganglia (Figure 1) [8,54]. GPR6 is an orphan receptor, but exhibits a constitutive activity coupled to a stimulatory Gs-protein and induces the increase in the intracellular cAMP levels. Initial studies reported that GPR6 is a lysophospholipid sphingosine 1-phosphate (S1P) receptor, but these results have not been confirmed by other groups [55,56]. GPR6 is involved in human instrumental learning in which the corticostriatal circuitry and the dopaminergic system participate [54,57]. The overexpression of GPR6 can promote neurite outgrowth in rat primary cerebellar granule neurons [58].

Oeckl *et al.* investigated neurochemical and behavioral phenotypes of GPR6 knockout mice [59]. GPR6 knockout mice show a reduction of striatal cAMP and an increase of dopamine, and exhibit higher locomotion activity. They reduce dyskinesia after apomorphine and quinpirole treatment in a mouse model of Parkinson’s disease, suggesting that the GPR6 inhibition may lead to improvement of Parkinson’s disease. A significant elevation in the phosphorylation of DARPP-32 (dopamine and cAMP-regulated phosphoprotein of 32 kDa) at Thr34 is detected in the GPR6 knockout mice, whereas DARPP-32 expression in the striatum of the GPR6 knockout mice was not altered. The phosphorylation of DARPP-32 at Thr34 by the cAMP/proteinkinase A (PKA) pathway plays a critical role in dopamine D1 and D2 receptor signal transduction [60]. It has been shown that the D2 receptor antagonist haloperidol increases DARPP-32 phosphorylation at Thr34 in striatopallidal neurons [61]. This finding could suggest that GPR6 also has the potential to be a therapeutic target for the treatment of schizophrenia.

### 5.4. GPR52

**Figure 2:**
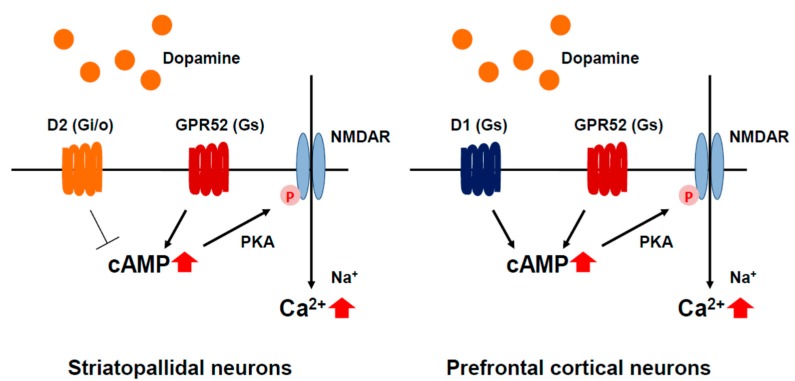
Proposed GPR52 signal transduction. GPR52 activation counteracts Gi/o-coupled D2 receptors in striatopallidal neurons (**left**); and potentiates NMDA activity through phosphorylation of the NMDA receptor via cAMP/PKA, as seen in D1 receptor-NMDA signal transduction (**right**).

GPR52 is an orphan GPCR, although the antipsychotic drug reserpine is identified as a surrogate ligand to induce intracellular cAMP accumulation and the receptor internalization, indicating that this receptor is a Gs-coupled receptor [8]. GPR52 is highly conserved among vertebrates with over 90% amino acid sequence identity, and is abundantly expressed in the brain, especially in striatum, with no apparent differences among species. More intriguingly, GPR52 shows a unique expression pattern where this receptor is expressed in almost all of the D2 expressing-MSNs of the basal ganglia while largely expressed in the D1 expressing neurons in the medial prefrontal cortex (Figure 1 and Figure 2). This raises the possibility that the GPR52 activation improves positive symptoms of schizophrenia by antagonizing the Gi/o-coupled D2 receptor activity in striatopallidal MSNs and improves schizophrenic negative symptoms and cognitive impairment through enhancement of the NMDA receptor activity via protein kinase A (PKA) in prefrontal cortical neurons, as seen in D1 receptor-NMDA signal transduction (Figure 2) [62,63,64].

The extensive analysis of the axonal projections of the GPR52-expressing neurons has shown that GPR52 is expressed in the limbic neural circuit necessary for recollection and spatial memory [65]. In addition, the GPR52-expressing neurons project from the habenular nucleus to the midbrain, where a negative reward signal in dopaminergic neurons originates [66]. In the prefrontal cortex, almost all of the GPR52-expressing neurons are glutamatergic, whereas only about 10% of the neurons are GABAergic [8]. Using hGPR52-GFP transgenic (Tg) mice in which the GFP (green fluorescent protein)-fused human GPR52 is functional *in vitro*, hGPR52-GFP and D2 receptor proteins are clearly divided around striatal regions. The GPR52-GFP is mainly seen in the lateral globus pallidus (LGP) whereas most of the D2 receptor proteins are localized in the striatum. This suggests that in the striatal neurons, GPR52 is transported to the axon terminals near the LGP, while the D2 receptor is localized in the dendritic spines [8].

Human GPR52 (hGPR52) Tg and GPR52 knockout (KO) mice have been generated and investigated so far [8]. The methamphetamine (MAP)-induced hyperlocomotion of hGPR52 Tg mice is more attenuated than that of non-Tg mice, whereas hGPR52 Tg mice show normal locomotor activity under normal conditions, suggesting that overexpression of GPR52 may counteract hyperdopaminergic transmission by MAP, a psychostimulant excessively releasing dopamine in the brain. In the open field test, GPR52 KO mice stay and move around the central zone longer, showing that they exhibit anxiolytic-like behavior. In addition, GPR52 KO mice are more sensitive to the startle response of the prepulse inhibition (PPI) test following the administration of NMDA receptor antagonist MK-801. Schizophrenia patients have impaired startle habituation and PPI of the startle reflex, and both typical and atypical antipsychotics ameliorate PPI deficits and diminishing startle response [67]. These findings indicate that GPR52 could modulate not only dopamine transmission but also NMDA signaling [8], hypofunction of which has been believed to cause symptoms of schizophrenia [68]. Recently, a potent and orally available GPR52 agonist with good pharmacokinetic properties has been reported [64]. This agonist significantly attenuates methamphetamine-induced hyperactivity in mice after oral administration of 3 mg/kg and shows a low risk of extrapyramidal symptoms (EPS), supporting the idea that the GPR52 activation counteracts active dopaminergic transmission in the limbic system. The elucidation of the molecular mechanism of GPR52 may open new avenues for the exploration of dopamine and NMDA systems, as well as the development of novel antipsychotic drugs.

## 6. Conclusions

Psychiatric disorders such as major depression, bipolar disorders, and schizophrenia bring about not only serious morbidity and mortality but also extraordinary economic burdens for patients’ families and society. In schizophrenia, for instance, the existing typical and atypical antipsychotics are not effective enough to ameliorate negative symptoms and cognitive impairment [69,70]. Despite strenuous efforts to develop innovative neuropharmacological drugs including antipsychotic agents, the pharmaceutical industries have so far failed to generate any drugs to fulfill an unmet medical need [71]. Thus, I believe that new therapeutic targets need to be identified for the treatment of mental illness. An increased understanding of the etiology of these illnesses as well as enormous biological data from various types of research should help in developing improved therapeutics. In this review, I have paid attention to the GPCRs that are enriched in the striatum as psychiatric therapeutic targets, as the striatum is a crucial brain region for psychiatric diseases. Besides psychoses, striatally enriched GPCRs such as GPR52 have been recently reported to modulate Huntington’s disease (HD) phenotypes both in the iPS-derived neurons of a patient and in the Drosophila HD models [72]. The GPCR expression data across many tissues in mice and humans (SymAtlas, SAGEmap) will aid in the rational use of animals to model GPCR function in psychiatric disorders and may help identify novel therapeutic targets and predict on-target side effects.
